# Metabolic Disorder of Extracellular Matrix Mediated by Decorin Upregulation Is Associated With Brain Arteriovenous Malformation Diffuseness

**DOI:** 10.3389/fnagi.2020.584839

**Published:** 2020-12-07

**Authors:** Maogui Li, Qingyuan Liu, Junhua Yang, Pengjun Jiang, Yi Yang, Yanan Zhang, Yong Cao, Jun Wu, Shuo Wang

**Affiliations:** ^1^Department of Neurosurgery, Beijing Tiantan Hospital, Capital Medical University, Beijing, China; ^2^China National Clinical Research Center for Neurological Diseases, Beijing, China; ^3^Center of Stroke, Beijing Institute for Brain Disorders, Beijing, China; ^4^Beijing Key Laboratory of Translational Medicine for Cerebrovascular Diseases, Beijing, China; ^5^Department of Blood Transfusion, Beijing Tiantan Hospital, Capital Medical University, Beijing, China

**Keywords:** brain arteriovenous malformation, diffuseness, extracellular matrix, DCN, TGF-beta pathway

## Abstract

**Background and Objective:**

Diffuse brain arteriovenous malformations (BAVMs) are mixed up with normal brain parenchyma and therefore increase the difficulty of surgical resection, leading to poor surgical prognosis. Since the mechanism underlying BAVM diffuseness remains unknown, a quantitative proteomic analysis was performed to investigate the altered expression of proteins in diffuse BAVMs compared to compact ones.

**Methods:**

We performed proteomic analysis on five diffuse BAVMs and five compact BAVMs. Bioinformatics analysis was conducted to identify potential signals related to BAVM diffuseness. Candidate proteins were then investigated in BAVM specimens using immunofluorescence and Western blot analysis. Tube formation assays were used to investigate the effects of candidate proteins on the angiogenesis of human umbilical endothelial cells (HUVECs). Finally, Masson, Sirius red staining, and immunofluorescence were used to evaluate the characteristics of extracellular matrix (ECM) in BAVM tissues.

**Results:**

A total of 58 proteins were found to be differentially expressed between diffuse and compact BAVMs via proteomic analysis. TGF-β (transforming growth factor-beta) signaling pathway, ECM–receptor pathway, relaxin signaling pathway, and several other pathways were associated with BAVM diffuseness. The TGF-β signaling pathway is associated with angiogenesis; the role of this pathway in the formation of diffuse BAVMs was investigated, and the decorin (DCN) upregulation played an important role in this process. Immunofluorescence showed that DCN was significantly upregulated within and around the malformed vessels of diffuse BAVMs. Functional assays showed that exogenous DCN could promote the tube formation ability of HUVECs through inhibiting the TGF-β signaling pathway and overproducing ECM. Histological staining demonstrated the overproduction of ECM in diffuse BAVMs.

**Conclusion:**

TGF-β signaling pathway inhibited by DCN in vascular endothelial cells is related to BAVM diffuseness. The metabolic disorder of ECM caused by DCN upregulation may significantly contribute to the formation of diffuse BAVMs.

## Introduction

Brain arteriovenous malformations (BAVMs) are characterized by the nidus of dysplastic connections between feeding arteries and draining veins (Moftakhar et al., 2009; Rangel-Castilla et al., 2014; Derdeyn et al., 2017). Most BAVMs have a compact architecture with little brain tissue within the nidus (Spears et al., 2006; Du et al., 2007; Hashimoto et al., 2007). However, in some rare cases classified as diffuse BAVMs (dBAVMs), there can be massive brain tissues interspersed among the malformed vessels (Chin et al., 1992; Spears et al., 2006; Du et al., 2007; Hashimoto et al., 2007). Previously, the features of dBAVMs have been well discussed for predicting the difficulty of surgical resection and poor surgical outcomes (Al-Shahi et al., 2002; Du et al., 2005, 2007; Spears et al., 2006).

Chin et al. (1992) firstly investigated the relationship between the radiological appearance and the histopathological features of dBAVMs and demonstrated the existing normal brain parenchyma among the malformed vessels. In another study, Du et al. revealed that dBAVMs are usually fed by the thin-walled and fragile arteries (Du et al., 2007). It was suggested that immature vascular formation and abnormal interaction between brain tissue and the vessels exist in dBAVMs. In a recent study, the somatic KRAS mutation was found in sporadic BAVMs and was thought to be involved in BAVM genesis and relative manifestations (Nikolaev et al., 2018). As one of the KRAS family, KRAS5 is supposed to activate related pathways, leading to the immature vascular formation and abnormal extracellular matrix (ECM) metabolism in BAVMs (Cheng and Nussinov, 2018). However, because of lacking relative studies, the mechanism underlying formation of dBAVMs remains unknown.

To date only a few proteomic studies on BAVMs have been performed; moreover, only the altered proteins in BAVMs versus normal arteries have been researched (Bicer et al., 2010; Simonian et al., 2017; Wang et al., 2017). In this study, the differentially expressed proteins between dBAVMs and compact BAVMs (cBAVMs) were identified using isobaric tags for relative and absolute quantification (iTRAQ) method, with the aim to demonstrate the pathological mechanism of vascular development in dBAVMs at the protein level.

## Materials and Methods

### Patients and Samples Preparation

Between January 2017 and April 2018, patients who underwent microsurgical resection of BAVMs in our institution were recruited. The exclusion criteria were (1) patients older than 60 years; (2) patients who have a history of non-surgical treatment such as radiosurgery and endovascular treatment; and (3) patients who have cerebral infarction or other cerebrovascular diseases such as arteriovenous fistula and cavernous hemangioma. The nidus types were divided into cBAVMs and dBAVMs. In this study, a dBAVM was defined as the nidus containing normal brain parenchyma interspersed among the malformed vessels (Chin et al., 1992; Du et al., 2007). Two experienced neurosurgeons (J.W. and P.J.) who were blind to the clinical data documented the type of the nidus according to T1-weighted, T2-weighted, and time-of-flight magnetic resonance angiography (MRA) images, and possible divergence was resolved by consulting with a senior researcher (S.W.). Clinical information including age, gender, and hemorrhage history were collected from the electronic medical record system. Radiological features including nidus size, location, deep venous drainage, and perforating artery supply were identified on MRA and digital subtraction angiography (DSA). The Spetzler–Martine (S-M) grade was calculated.

The BAVM samples were obtained during surgery after total resection. All visible brain tissue and blood clot were removed, and only the malformed vessels were collected. Besides, the superficial temporal arteries (STA) of some patients (cases 1, 2, 3, 5, 8, and 9) were collected. Subsequently, each tissue sample was washed with low-temperature phosphate-buffered saline (PBS) in order to remove the blood cells within the vessels.

### Isobaric Tags for Relative and Absolute Quantification (iTRAQ) Labeling and Nano-LC-MS/MS Analysis

Frozen tissue samples were homogenized with RIPA buffer (Sigma-Aldrich, United States) by hand. The lysis buffers were placed on ice for 30 min and then centrifuged for 20 min under 15,000 *g* to remove insoluble components. The concentration of the extracted protein was quantitatively analyzed using the Bicinchoninic acid method (BCA method). One hundred fifty micrograms from each sample was alkylated and digested in the centrifugal unit. Then, the protein of each pool was dissolved with 1 M DTT for 1 h at 37°C and kept in the dark with 1 M indole-3-acetic acid for 1 h at ambient temperature after precipitating with acetone. Samples were dissolved and centrifuged twice with 120 μl of UA (8 M urea in 0.1 M Tris–HCl, pH 8.5) and then re-dissolved and centrifuged three times with 100 mM lautyltrethylammonium bromide. The proteins were digested with trypsin (Sigma-Aldrich, United States) and incubated at 37°C overnight. Subsequently, each peptide pool was passed through a 0.2-μm centrifugal filter for 20 min under 12,000 *g* at 20°C. Prepared peptide samples were labeled using a 4-Plex iTRAQ Reagent Kit from AB SCIEX. cBAVMs, dBAVMs, and STAs were labeled with iTRAQ tags, respectively. The labeled peptide mixtures were separated and then trapped on a PepMap100 C18, 5 μm, 100 μm × 2 cm column (Thermo Scientific, United States) using the EASY-nLC 1000 system (Thermo Scientific, United States). Subsequently, the peptides were separated on a PepMap100 RSLC C18, 2.4 μm, 75 μm × 15 cm analytic column by a 102-min mobile phase gradient (from 5 to 90%). Spectra were recorded by the Orbitrap Elite system (Thermo Scientific, United States). Full scan MS spectra were obtained in the *m*/*z* range 400–1600 at a resolution of 60,000; the top precursors were selected for high-energy collision-induced dissociation with a collision energy of 35%; the product ions were detected at a resolution of 15,000 by Data Dependent Analysis.

### Bioinformatics Analysis

First, the raw data were searched and discovered using Proteome Discoverer 1.4 (Thermo Scientific, United States). Subsequently, we excluded the proteins with unique peptide as 0 or described as false-positive proteins in the Decoy database. Next, the altered proteins with *P* value < 0.05 and fold value ≥2 or ≤0.5 between dBAVMs and cBAVMs were selected (selected proteins). Then, the proteins whose difference was not obvious between dBAVMs and STAs (fold value was between 0.5 and 2.0) were excluded from the selected proteins; meanwhile, the remaining proteins were summarized as a new altered protein database. Besides, CytoScape 3.6.1 (a free software) was used for further bioinformatics analysis. According to this database, pathway enrichment analysis was performed based on the Kyoto Encyclopedia of Genes and Genomes (KEGG) database using ClueGo (a free plug-in in CytoScape). Gene Ontology (GO) database was employed for the biological interpretation of the identified protein using ClueGo. The differentially expressed proteins of GO were stratified into two types, namely, molecular function and cellular component. The function interaction between different pathways was conducted based on the REACTOME database. The protein–protein interaction (PPI) analysis was conducted based on the STRING database.

### Western Blot Analysis

Besides the 10 BAVM samples for iTRAQ analysis, another 6 BAVM samples were also collected for further validation. Equal amounts of protein were separated by SDS-PAGE and then electro-transferred to PVDF membranes. Subsequently, membranes were blocked with the PBST containing 5% bovine serum album (Sigma-Aldrich, United States) for 1 h and then probed with primary antibodies at 4°C overnight, including rabbit anti-DCN antibody (Abcam, United Kingdom) at 1:1000, rabbit anti-Smad2/3 antibody (Abcam, United Kingdom) at 1:100, rabbit anti-Col I antibody (Abcam, United Kingdom) at 1:1000, and rabbit anti-GAPDH antibody (Abclonal Technology, Wuhan, China). After primary incubation and being washed with PBST, membranes were incubated with secondary antibodies at 1:5000 for 1 h at room temperature. Bands were visualized by an ECL detection system (GeneSys, Alcatel, France). The expression levels were quantified with ImageJ (version 1.8.0, a free software). The expression levels of target proteins were evaluated by performing densitometric analysis. Ratios of target protein densitometric measurements to GAPDH were used for further analysis.

### Histological Staining

We performed histological staining in all 16 BAVM samples. The samples with appropriate volume were taken before dehydration, fixation, and paraffin embedding. For histology, sections were stained with H-E (Hematoxylin–Eosin), Masson, and Sirius red kit (Solarbio, China). For immunofluorescence (IF), sections were blocked with Protein block (Abcam, United Kingdom) after antigen retrieval and permeabilization. Then, sections were incubated with primary antibodies at 4°C overnight, including rabbit anti-DCN (Abcam, United Kingdom) at 1:100, rabbit anti-Smad 2/3 (Abcam, United Kingdom) at 1:100, rabbit anti-Col I antibody (Abcam, United Kingdom) at 1:500, rabbit anti-Col III antibody (Abcam, United Kingdom) at 1:1000, and rabbit anti-Col VI antibody (Abcam, United Kingdom) at 1:500. After being washed with PBST, sections were incubated with secondary antibody (Alexa Fluor^®^ 488 Goat anti-rabbit IgG, Abcam, United Kingdom, and Alexa Fluor^®^ 647 Goat anti-rabbit IgG, Abcam, United Kingdom) at 1:500. Subsequently, sections were stained with DAPI (4′,6-diamidino-2-phenylindole, Solarbio, China) after being washed with PBST. Besides, a laser confocal microscopy workstation (LSM 710, ZEISS, Germany) was used to capture images. Identical conditions and set integration times were applied to facilitate comparisons between samples.

The Masson staining was used to detect fibrosis in BAVMs. Three random sections were measured by two investigators (Q.L. and J.Y.) using ImageJ blinded to the type of BAVMs. The fibers were stained blue, and the non-fibrotic area was stained red. We defined the fibrotic area as the blue area/(blue area + red area). As for IF, ImageJ was used to calculate the integrated optical density (IOD) and count the cell number based on the DAPI image. We used the IOD/cell for further analysis.

### Cell Culture and Decorin Treatment

The human umbilical vein endothelial cells (HUVECs) were purchased from ScienCell (Carlsbad, CA) and maintained in endothelial cell medium (ScienCell corporation) supplemented with 5% fetal bovine serum (Gibco), 100 U/ml penicillin, and 100 μg/ml streptomycin. We used Recombinant Human Decorin Protein (1 μg/ml, R&D Corporation) to treat HUVECs for 24 h. After appropriate treatment, the cells were used for further analysis.

### Tube Formation Assay

Tube formation assays were performed using Ibidi μ-Slide angiogenesis (Ibidi Corporation) according to the manufacturer’s protocol. A total of 1.5 × 10^4^ HUVECs in 50 μl of complete media were planted with Matrigel. The slides were subsequently incubated at 37°C for 24 h. The tube formation was observed using the Fluorescence Inversion Microscope system, and ImageJ was used to calculate the number of meshes.

### Statistical Analysis

All statistical analyses were conducted using GraphPad Prism 5 (GraphPad Software, American). Variables were compared by the χ2 test, Fisher’s exact test, independent Student’s *t* test, or Mann–Whitney *U* test. *P* < 0.05 was considered to be statistically significant.

## Results

### Demographic and Clinical Characteristics of the Study Population

A total of 10 patients with BAVMs were recruited in this study, including 5 dBAVMs and 5 cBAVMs (cases 1–10, see Supplementary Figure 1). The clinical information of all cases is listed in Table 1. Five patients were female and five were males, with ages ranging from 16 to 41 years. The average diameter of nidus was 4.81 cm (ranged from 3.71 to 5.69 cm). No significant difference was found in age, sex, hemorrhage history, nidus size, and S-M grade between the dBAVMs group and cBAVMs group. Three patients who suffered from acute BAVM rupture received early surgical treatment within 3 days after admission. The S-M grade of these BAVMs was only II or III, because of the limited chances of obtaining samples from grade I BAVMs and the rare surgical cases of grade IV or V.

**TABLE 1:** The demographic and clinical information of BAVM patients.

**Characteristics**	**cBAVMs *n* = 5**	**dBAVMs *n* = 5**	***P* value**
Age, years	29.3 ± 11.0	24.5 ± 8.1	0.548
Male, n (%)	2	3	0.690
Hemorrhage history, *n* (%)	2	1	0.690
Locations, *n* (%)			0.548
Temporal	3	2	
Frontal	2	2	
Occipital	0	1	
Size, cm	5.2 ± 0.6	4.5 ± 0.8	0.841
Deep venous drainage, *n* (%)	3	3	1.000
Perforating artery supply, *n* (%)	2	3	0690
Spetzler–Martin grade, *n* (%)			1.000
2	1	1	
3	4	4	

*cBAVMs, compact brain arteriovenous malformations; dBAVMs, diffuse brain arteriovenous malformations.*

### TGF-β Signaling Pathway Was Associated With BAVM Diffuseness

**FIGURE 1:**
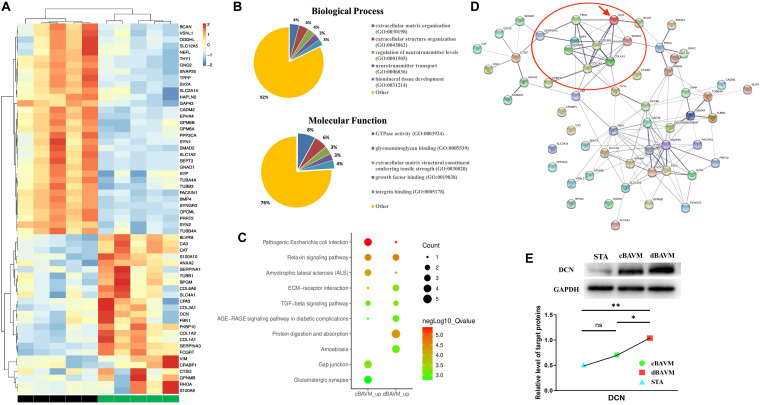
Proteome profiles of diffuse and compact brain arteriovenous malformations (BAVMs). **(A)** Protein expression levels in diffuse and compact BAVMs (*P* ≤ 0.05, fold change ≥2 or ≤0.5, and excluded the proteins with fold value of 0.5 to 2.0 between diffuse BAVMs and superficial temporal arteries). **(B)** The function and biological process enriched by altered protein by Gene Ontology (GO) analysis. **(C)** Top 10 pathways enriched by protein upregulation in diffuse and compact BAVMs by Kyoto Encyclopedia of Genes and Genomes (KEGG) analysis. **(D)** The protein–protein interaction network of altered proteins by STRING analysis; the decorin (DCN) was highlighted by a red arrow. The proteins interacted with DCN were highlighted by a red circle. **(E)** Validation of DCN expression among superficial temporal arteries, compact BAVMs, and diffuse BAVMs. A group of representative cases from each sample is shown; moreover, the level of DCN in each sample was given in Supplementary Figure 3. ^∗^*P* < 0.05; ^∗∗^*P* < 0.01; ns, *P* > 0.05. STA, superficial temporal arteries; cBAVMs, compact brain arteriovenous malformations; dBAVMs, diffuse brain arteriovenous malformations.

Proteomic changes among the samples of five dBAVMs, five cBAVMs, and six STAs were assessed using iTRAQ analysis. A total of 84 significantly altered proteins were identified (defined as fold value ≥2 or ≤0.5 and a *P* value < 0.05) from 3080 proteins. Twenty-six proteins were then excluded for no significance between BAVMs and STAs (defined as fold value of 0.5 to 2.0). Of the 58 altered proteins (see Figure 1A and Supplementary Table 1), 33 proteins were upregulated, and 25 proteins were downregulated (the Top 10 upregulated and downregulated proteins are listed in Tables 2, 3, respectively).

**TABLE 2:** Top 10 proteins significantly upregulated in dBAVMs comparing with cBAVMs.

**No.**	**Protein**	**Accession no.**	**Gene name**	**Protein function**	**Fold change**
1	Mast cell carboxypeptidase A	CBPA3	CPA3	Metallocarboxypeptidase activity	2.43
2	Alpha-1-antichymotrypsin	AACT	SERPINA3	Serine-type endopeptidase inhibitor activity	2.42
3	Collagen alpha-2(I) chain	A0A087WTA8	COL1A2	ECM structural constituent	2.38
4	Collagen alpha-1(I) chain	CO1A1	COL1A1	ECM structural constituent	2.36
5	Protein S100-A10	S10AA	S100A10	Calcium ion binding	2.31
6	Collagen alpha-6(VI) chain	CO6A6	COL6A6	ECM structural constituent	2.29
7	Tubulin beta-1 chain	TBB1	TUBB1	Structural constituent of cytoskeleton	2.28
8	Collagen alpha-1(III) chain	CO3A1	COL3A1	ECM structural constituent	2.26
9	Decorin	DCN	PGS2	ECM structural constituent	2.20
10	Rho-related GTP-binding protein	RHOA	RHOA	Protein binding	2.20

*ECM, extracellular matrix.*

**TABLE 3:** Top 10 proteins significantly downregulated in dBAVMs compared with cBAVMs.

**No.**	**Protein**	**Accession no.**	**Gene name**	**Protein function**	**Fold change**
1	Proline-rich transmembrane protein 2	PRRT2	PRRT2	Protein binding	0.34
2	Ephrin type-A receptor 4	E9PG71	EPHA4	ATP binding	0.37
3	Cell adhesion molecule 2	CADM2	IGSF4D	Cell adhesion	0.38
4	Neuronal membrane glycoprotein M6-a	GPM6A	M6A	Calcium channel activity	0.39
5	Hyaluronan and proteoglycan link protein 2	Q9GZV7	HPLN2	Hyaluronic acid binding	0.39
6	Guanine nucleotide-binding protein G subunit alpha	GNAO	GNAO1	Protein binding	0.41
7	Tubulin beta-3 chain	TBB3	TUBB4	Structural constituent of cytoskeleton	0.41
8	Excitatory amino acid transporter 2	EAA2	EAAT2	Glutamate: sodium symporter activity	0.41
9	Synaptic vesicle glycoprotein 2A	SV2A	KIAA0736	Protein binding	0.41
10	Visinin-like protein 1	VISL1	VISL1	Calcium ion binding	0.43

An enrichment analysis was performed to elucidate the functional implications of the altered proteins. Based on the GO database, most of the altered proteins were located in the ECM, presynapse, and axon part; moreover, the molecular function of altered proteins included glycosaminoglycan binding and ECM structural constituent conferring tensile strength (see Figure 1B). The analysis based on REACTOME database showed that altered proteins were mainly clustered in the degradation of the ECM, microtubule-dependent trafficking of connexons from Golgi to the plasma membrane, Serotonin Neurotransmitter Release cycle, and Ca2 + pathway (see Supplementary Figure 2A). Therefore, we supposed that the metabolism of ECM might play an important role in the formation of dBAVMs, which was determined to be the focus of our next analysis. The analysis based on KEGG database showed several clusters related to the TGF-β (transformation growth factor-beta) signaling pathway, ECM–receptor pathway, protein digest and absorption, relaxin signaling pathways, and gap junction (see Table 4 and Figure 1C). The ECM proteins (CO6A6, CO3A1, and so on) mainly enriches in ECM–receptor pathway, relaxin signaling pathway, and protein digestion and absorption pathway. Our further analysis showed that the TGF-β signaling pathway could interact with the ECM–receptor pathway and relaxin signaling pathway (see Supplementary Figure 2B). TGF-β signaling pathway is associated with angiogenesis and metabolism of ECM and is related to the diffuseness of BAVMs. Although our analysis also confirmed the role of Relaxin signaling pathway, this pathway mainly regulates the process of post-injury healing, vasoconstriction, and inflammation (Dschietzig et al., 2012; Moon et al., 2014; Valle Raleigh et al., 2017), which is related to atherosclerosis, bone formation, and so on. During the process of angiogenesis, the TGF-β signaling pathway could regulate the endothelial cell and the metabolism of ECM. Among the protein enriched in the TGF-β signaling pathway, the decorin (DCN) was identified as the most strongly altered protein (see Supplementary Figure 2C). DCN interacts with some major component of ECM, which suggests that DCN may play an important role in metabolism of ECM in BAVMs (see Figure 1D). The expression of DCN in dBAVMs, cBAVMs, and STA was found to be significantly differential by Western blot (see Figure 1E and Supplementary Figure 3). The level of TGF-β was not significant between cBAVMs and dBAVMs (Supplementary Figure 3). These results suggested that inhibition of TGF-β signaling pathway was associated with BAVM diffuseness, and DCN might play an important role in this pathological process.

**TABLE 4:** Top 10 enriched pathways identified by KEGG analysis.

**No.**	**Term**	**Count**	**ID**	***P* value**
1	Relaxin signaling pathway	6	hsa04926	<0.001
	Protein digestion and absorption	5	hsa04974	<0.001
2	Pathogenic *Escherichia coli* infection	5	hsa05130	<0.001
3	TGF-beta signaling pathway	4	hsa04724	<0.001
4	ECM–receptor interaction	4	hsa04512	<0.001
5	Gap junction	4	hsa04540	<0.001
6	AGE-RAGE signaling pathway in diabetic complication	4	hsa04933	<0.001
7	Amyotrophic lateral sclerosis (ALS)	4	hsa05014	<0.001
8	Amoebiasis	4	hsa05146	<0.001
10	Glutamatergic synapse	4	hsa04724	<0.001

### DCN Was Upregulated in Malformed Vessels and Might Regulate the Expression of Smad 2/3

**FIGURE 2:**
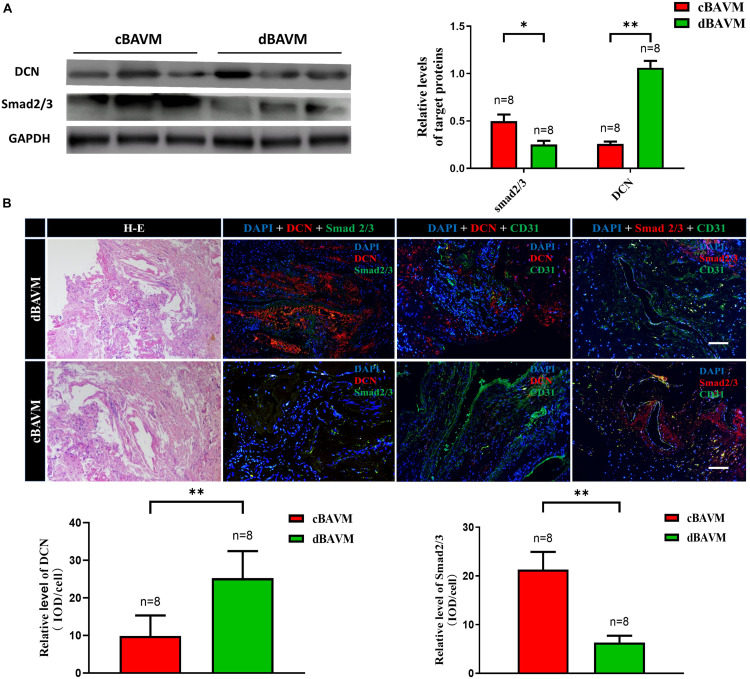
The expression level of DCN and Smad 2/3 in BAVM tissues. **(A)** Western blot analysis further validated the upregulation of DCN and downregulation of Smad 2/3 (Smad family member 2 and 3) in another six BAVM tissues (three cBAVMs and three dBAVMs; the information of these six BAVM patients was given in Supplementary Table 2). ^∗^*P* < 0.05; ^∗∗^*P* < 0.01. **(B)** Histological staining was performed in all 16 BAVM samples. Two groups of representative images of hematoxylin–eosin (H-E) and immunofluorescence staining for DCN, Smad 2/3, and CD31 in BAVM tissues. The histograms showed the semiquantitative grading of DCN and Smad 2/3 expression levels in diffuse and compact BAVMs. The scale bar corresponds to 200 μm. ^∗∗^*P* < 0.01.

To further validate the differential expression of DCN between cBAVMs and dBAVMs, and to determine the distribution characteristics of DCN, we performed IF and Western blot. The information of another six patients was given in the Supplementary Table 2. For another six BAVMs, the Western blot showed that DCN was upregulated but Smad 2/3 were downregulated in dBAVMs (see Figure 2A). For all 16 BAVMs, the IF showed that DCN was mainly detected in and around malformed vessels and was upregulated in dBAVMs (*P* < 0.001); moreover, as was mainly expressed by vascular endothelial cells (which were marked by CD31), Smad 2/3 was downregulated as the DCN increased in dBAVMs (see Figure 2B).

### DCN Upregulation Promoted Endothelial Cell Tube Formation by Regulating the Production of ECM

**FIGURE 3:**
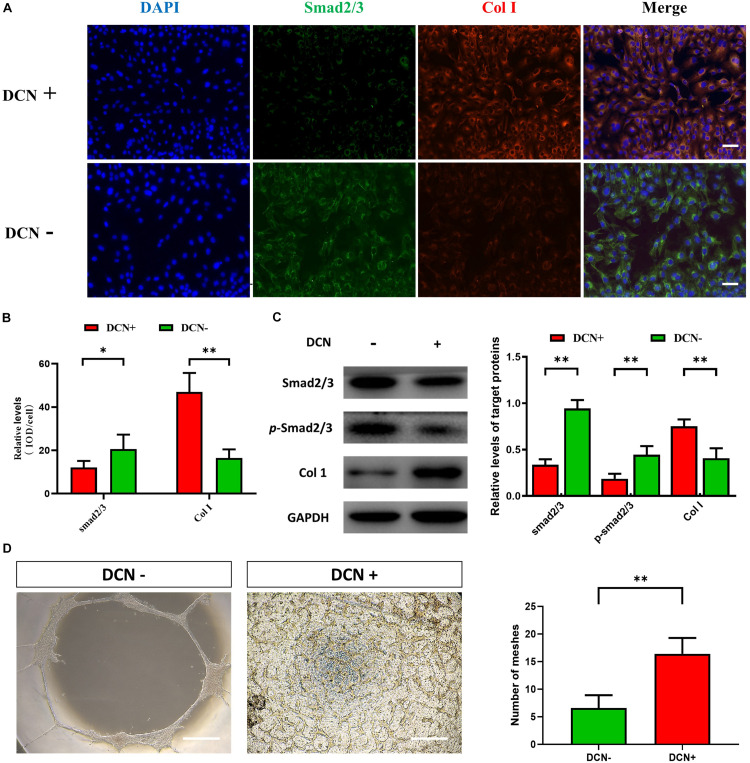
The effect of DCN upregulation on angiogenesis for human umbilical vascular endothelial cells (HUVECs). **(A)** Immunofluorescence staining for HUVECs with and without DCN treatment. One representative experiment of 3 is shown. The scale bar corresponds to 50 μm. **(B)** The histograms showed the semiquantitative grading of DCN and Smad 2/3 expression levels by immunofluorescence staining in HUVECs with and without DCN treatment. ^∗^*P* < 0.05; ^∗∗^*P* < 0.01. **(C)** Western blot analysis of total Smad 2/3, phosphorylated Smad 2/3 (p-Smad 2/3), and Col I in HUVECs with and without DCN treatment. One representative experiment of 3 is shown. The histograms showed the semiquantitative grading of total Smad 2/3, p-Smad 2/3, and Col I expression levels in HUVECs with and without DCN treatment. ^∗∗^*P* < 0.01. **(D)** Tube formation assays after different treatment. The scale bar corresponds to 50 μm. One representative experiment is shown. ^∗∗^*P* < 0.01.

As an extracellular protein, DCN can interact with transforming growth factor (TGF) to inhibit the TGF-β pathways. To investigate the effect of DCN on TGF-β pathway and the role of DCN in angiogenesis, we added exogenous DCN (1 μg/ml) to HUVEC. After stimulating the HUVEC by DCN, both IF and Western blot confirmed the downregulation of Smad 2/3 and upregulation of Col I (see Figures 3A–C). ECM includes collagen, laminin, fibronectin, and proteoglycans. In this study, we mainly detected the expression of Col I, which is the major component of collagen. Smad 2/3 is a cell-intrinsic regulator of TGF-β pathway and can affect the process of angiogenesis; also, Col I is associated with angiogenesis.

As DCN was upregulated in dBAVMs, we hypothesized that DCN could promote the angiogenesis by stimulating the overproduction of ECM. After DCN stimulation, phosphorylated Smad 2/3 was significantly downregulated in HUVECs compared to the HUVECs without DCN treatment. Moreover, further study showed that the tube formation of HUVECs was enhanced after treatment of DCN (see Figure 3D). Collectively, these results showed that DCN could promote the endothelial cell tube formation by inhibiting the TGF-β pathway and stimulating the overproduction of ECM.

### Metabolic Disorder of ECM Might Promote BAVM Diffuseness

**FIGURE 4:**
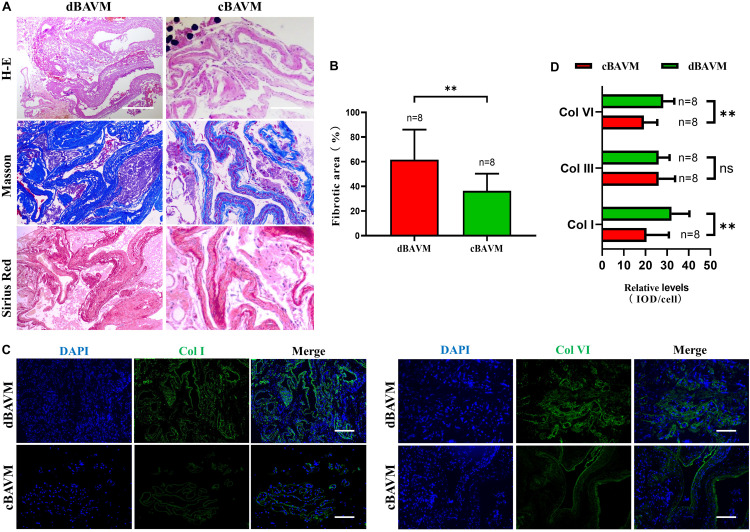
Upregulation of DCN promotes the overproduction of extracellular matrix in diffuse BAVMs. **(A)** Masson and Sirius red staining for collagen deposition in BAVM tissues. Fibronectin and collagen area indicated as blue. The scale bar corresponds to 200 μm. ^∗∗^*P* < 0.01. **(B)** The histograms showed the semiquantitative grading of fibrotic area in diffuse and compact BAVMs. **(C)** Immunofluorescence staining for Col I and Col VI in BAVM tissues. Several representative images of diffuse and compact BAVMs are shown. The scale bar corresponds to 200 μm. **(D)** The histograms showed the semiquantitative grading of Col I, Col III, and Col VI in diffuse and compact BAVMs. ^∗∗^*P* < 0.01; ns, *P* > 0.05.

To further investigate the composition and characteristics of ECM in cBAVMs and dBAVMs, we performed histological staining in all the 10 BAVMs. The results from the Masson staining showed an obvious vascular fibrosis in cBAVMs compared with dBAVMs; moreover, the Sirius red staining showed a larger area of collagen I and less area of other type of collagen (see Figures 4A,B). In proteomic analysis, the result showed that only collagen I, III, and VI might be significantly altered between the two groups. Subsequently, we performed IFs to detect the differential expression of collagen I, III, and VI between cBAVMs and dBAVMs. Our findings showed that collagen I and collagen VI were both significantly upregulated in dBAVMs (see Figures 4C,D). These results demonstrated the dysregulation of ECM in dBAVMs. The abnormal metabolism of collagen I and collagen VI may lead to BAVM diffuseness during the process of angiogenesis.

## Discussion

Although previous studies revealed several mechanisms underlying BAVM genesis and rupture (Moftakhar et al., 2009; Rangel-Castilla et al., 2014; Wang et al., 2017), the forming mechanisms of dBAVMs remain unknown. Studying the altered molecular of dBAVMs contributes to understanding the vascular dysplasia in BAVMs and promoting the possibility of medical treatment. In this study, we performed a proteomics study using the iTRAQ method and revealed that metabolic disorder mediated by DCN might play an important role in the formation of dBAVMs.

In this study, after excluding similar proteins between BAVMs and normal STAs, we found that there were a total of 58 significantly altered proteins, including 33 upregulated and 25 downregulated in dBAVMs compared with cBAVMs. Interestingly, the altered proteins between dBAVMs and cBAVMs are mainly involved in the ECM structural constituent and metabolism of ECM, suggesting that the composition of ECM may be different between different types of BAVMs. Moreover, the majority of enriched pathways take part in regulating the metabolism of ECM and interact with the TGF-β signaling pathway. Previous studies found that TGF-β signaling pathway plays an important role in the formation and clinical features of BAVMs (Wang et al., 2018; Fu et al., 2020). According to the classical theory of angiogenesis, the formation of blood vessels consists of two critical parts, vasculogenesis and angiogenesis, which are induced by the orchestra effect of vascular cells and ECM (Hanahan, 1997; Rangel-Castilla et al., 2014). Normal metabolism of ECM is important for guaranteeing the process of angiogenesis (Rangel-Castilla et al., 2014). Notably, in addition to TGF-β pathway, we found the high expression of collagen I, III, and VI (enriched in the ECM receptor pathway) in dBAVMs, suggesting an overproduction of ECM in dBAVMs compared to cBAVMs. Therefore, we supposed that the metabolic disorder of ECM, presenting as overproduction of and less degradation of ECM, inhibits the formation of connections between vascular trunks, leading to the malformed vessels to be immature, scattered, and mixed up with brain tissues in dBAVMs.

The analyses based on the REACTOME and KEGG database implied that the pathways related to metabolism of ECM might play important roles in formation of dBAVMs. Previous studies identified that the ECM–receptor interaction pathway was important for BAVM genesis and development (Seker et al., 2006; Wang et al., 2017). In another study, it was found that the overgrowth of vessels activated by angiogenic factors might cause an incomplete vascular structure containing massive collagen (Hashimoto et al., 2005). Interestingly, after analyzing the interaction among pathways, we found that the ECM–receptor pathway was interacted with the TGF-β pathway. Considering the TGF-β signaling pathway is associated with angiogenesis and metabolism of ECM, we suggest that the dysregulation of TGF-β signaling pathways can lead to metabolic disorder of ECM, which may cause excessive proliferation of vasculature and immature vascular development, resulting in tiny vessels scattered within normal brain tissues. Based on these findings, we further investigate the expression level of proteins in the TGF-β pathway and found that DCN was the most strongly altered protein in dBAVMs compared to cBAVMs. Encoded by PGS2, DCN is a small dermatan sulfate proteoglycan that can interact with a variety of proteins associated with ECM assembly (Hocking et al., 1998). Several biological functions including cell migration, proliferation, and angiogenesis may be regulated by this molecule (Nash et al., 1999; Merle et al., 2015a,b). The biological effect of DCN is primarily suppressive for cell proliferation but promotive for ECM production, especially for collagen fibrils (Jarvelainen et al., 1991; Nili et al., 2003; Salomaki et al., 2008). Thus, we hypothesized that the metabolic disorder of ECM in dBAVMs might be mediated by DCN.

Based on this assumption, we further validated the expression level of DCN and Smad 2/3 (a cell-intrinsic regulator of TGF-β pathway) in BAVM tissues. It was shown that DCN was upregulated and Smad 2/3 was downregulated in dBAVMs; moreover, the DCN was found within and around the malformed vessels, and Smad 2/3 was mainly detected in vascular endothelial cells. In addition, the expression of COL I was upregulated in DCN treatment group. In further *vitro* study, we used exogenous DCN to treat HUVECs. The result showed that the expression of Smad 2/3 was inhibited after DCN treatment. Both Western-blot and IF suggested that DCN could inhibit TGF-β pathway and stimulate the expression of ECM, which was consistent with previous studies (Yan et al., 2009; Cabello-Verrugio et al., 2012). In a recent study, Jiang et al. found that DCN could interfere with the interaction of TGF-β and TGF receptor and promote ECM synthesis (Jiang et al., 2020). This pathological process could promote the angiogenesis of vascular endothelial cell, which was further verified by further functional assays where the ability of tube formation was enhanced after being treated by DCN. Subsequently, we determined the existing metabolic disorder in dBAVMs, presenting as overproduction of collagen, especially collagen I and VI. Collectively, the metabolic disorder of ECM mediated by DCN may play an important role in the formation of dBAVMs. The expression level of DCN is associated with the formation of dBAVMs, which suggests that the level of DCN in serum may be served as a non-invasive and quick biomarker to identify development of BAVM diffuseness. We suppose that evaluating and correcting the expression level of DCN may contribute insights into the diagnosis and treatment of dBAVMs. Future *in vivo* experiments may help to verify our hypothesis.

## Limitations

There were several limitations in the present study. First, due to the difficulty of collecting BAVM samples, the proteomic analysis based on limited samples might compromise the power of the results. Meanwhile, selection bias may exist in the present study, since we could not collect the STA samples of every patient for the limited scope of surgery. Second, the confounding effects of blood clot could not be completely excluded because the molecules such as S100A10 were also involved in clot formation (Seker et al., 2006). Nevertheless, since most of these molecules were involved in the metabolism of ECM, we thought that these molecules largely contributed to the metabolism of ECM. Finally, the other proteins and pathways identified in this work still need to be studied by further studies, since there may be other potential mechanisms that were not discussed in this study.

## Conclusion

This study is the first to explore the molecular mechanisms of BAVM diffuseness at the protein level. In total, 58 significantly altered proteins were identified in dBAVMs compared to cBAVMs. These altered proteins were primarily enriched in pathways related to the metabolism of ECM. TGF-β signaling pathway inhibited by DCN in vascular endothelial cells is related to BAVM diffuseness. The metabolic disorder of ECM induced by DCN upregulation significantly contributed to the formation of diffuse BAVMs. The definite association between DCN and dBAVMs needs to be investigated by further *in vivo* experiments. Our findings provide extra insights into understanding the mechanisms and promoting the possibility of medical treatment of dBAVMs.

## Data Availability Statement

The raw data supporting the conclusions of this article will be made available by the authors, without undue reservation.

## Ethics Statement

The studies involving human participants were reviewed and approved by the Institutional Review Board of Beijing Tiantan Hospital. The patients/participants provided their written informed consent to participate in this study.

## Author Contributions

SW is the principal investigator of this study and obtained the research funding, approved publication of this final manuscript. ML and QL have developed this manuscript. QL and JY revised this manuscript. PJ, YY, YZ, and YC provided assistant in experiments and statistics. All authors contributed to the article and approved the submitted version.

## Conflict of Interest

The authors declare that the research was conducted in the absence of any commercial or financial relationships that could be construed as a potential conflict of interest.
