# Supplementary figures and images for: Metabolic Disorder of Extracellular Matrix Mediated by Decorin Upregulation Is Associated With Brain Arteriovenous Malformation Diffuseness
Source: Front Aging Neurosci. 2020 Dec 7;12:584839. doi: 10.3389/fnagi.2020.584839 (PMC7750526; doi:10.3389/fnagi.2020.584839)

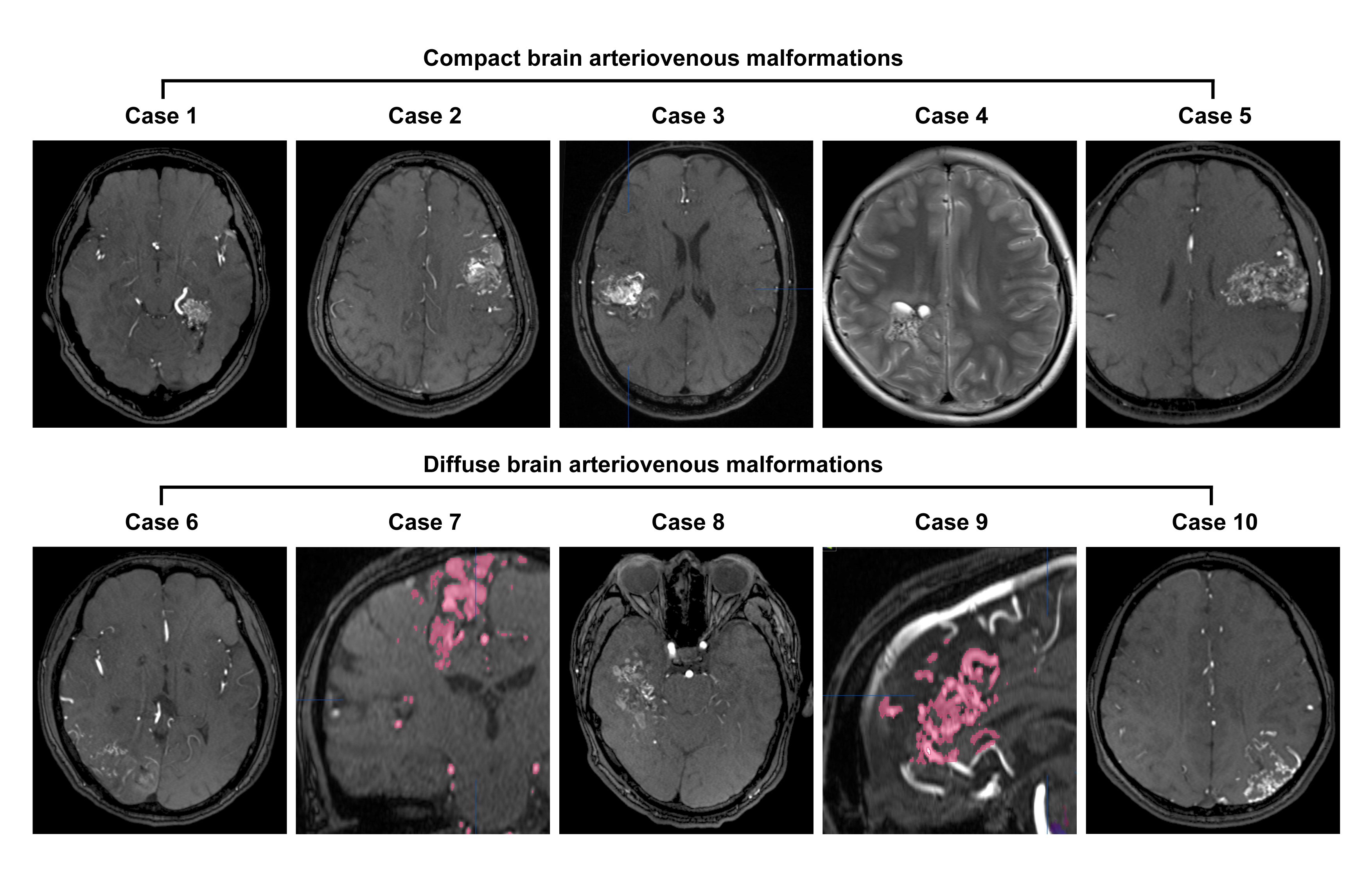

Supplement: Supplementary file 1 [file Image_1.jpeg]

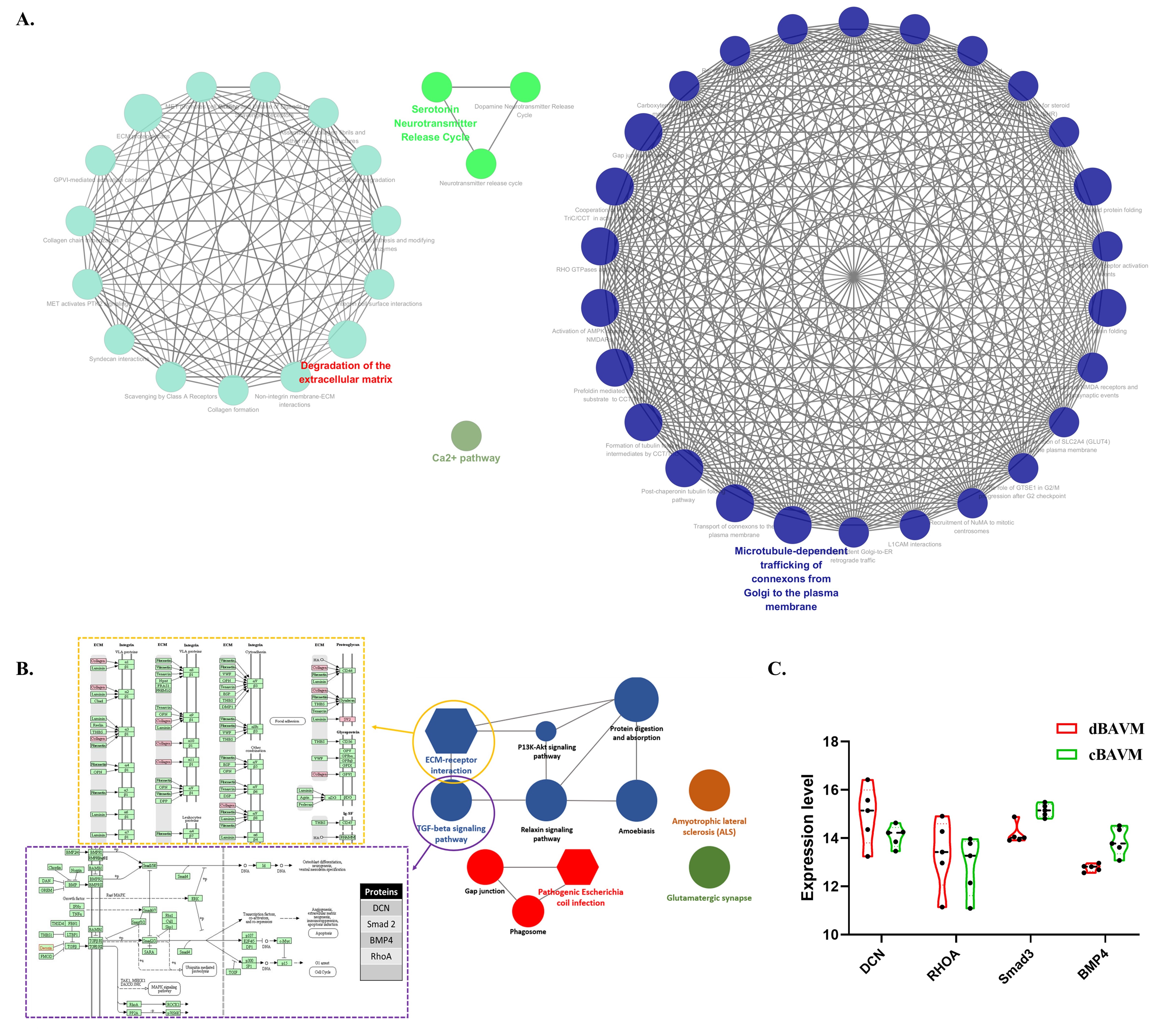

Supplement: Supplementary file 2 [file Image_2.JPEG]

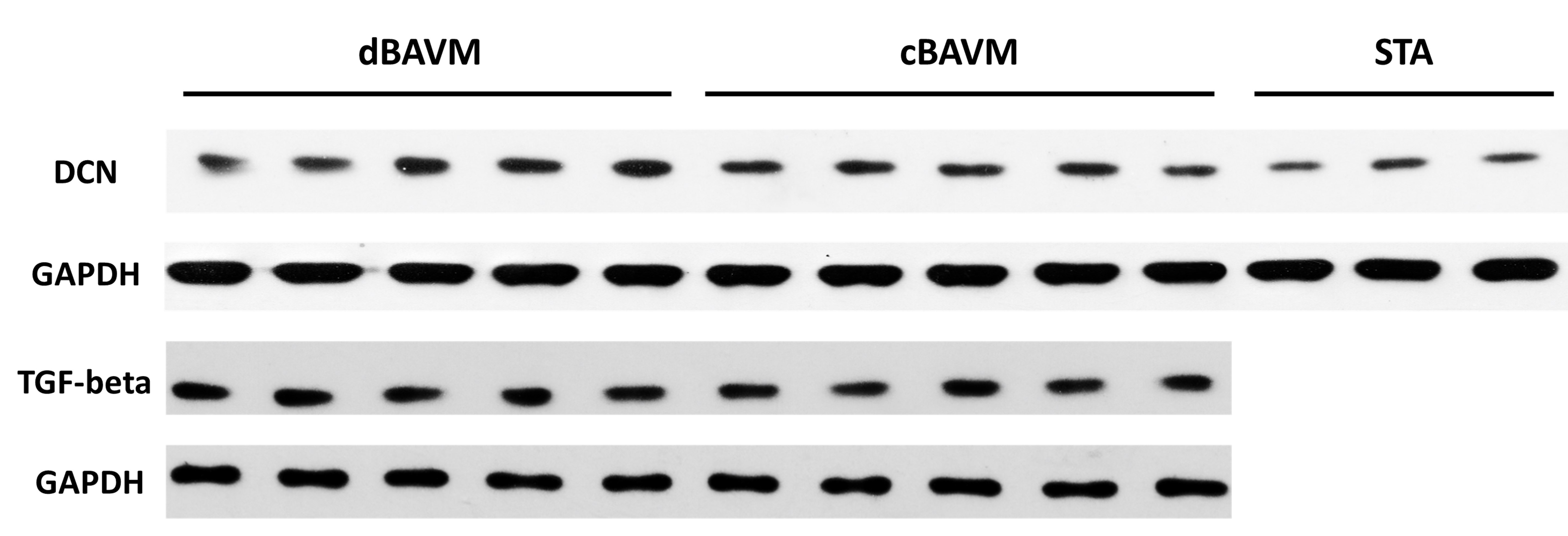

Supplement: Supplementary file 3 [file Image_3.JPEG]
