# Supplementary material for: Metabolic Disorder of Extracellular Matrix Mediated by Decorin Upregulation Is Associated With Brain Arteriovenous Malformation Diffuseness
Source: Front Aging Neurosci. 2020 Dec 7;12:584839. doi: 10.3389/fnagi.2020.584839 (PMC7750526; doi:10.3389/fnagi.2020.584839)
Supplement: Supplementary file 4 [file Table_1.docx]

| Supplementary Table 1 | |  |  |  |  |
| --- | --- | --- | --- | --- | --- |
| Significantly altered protein in dBAVMs to cBAVMs | | | |  |  |
| No. | Protein | Accession no. | Gene name | Fold change | *P* value |
| 1 | Mast cell carboxypeptidase A | CBPA3 | CPA3 | 2.43 | 0.0079 |
| 2 | Alpha-1-antichymotrypsin | AACT | SERPINA3 | 2.42 | < 0.0001 |
| 3 | Collagen alpha-2(I) chain | A0A087WTA8 | COL1A2 | 2.38 | < 0.0001 |
| 4 | Collagen alpha-1(I) chain | CO1A1 | COL1A1 | 2.36 | < 0.0001 |
| 5 | Protein S100-A10 | S10AA | S100A10 | 2.31 | < 0.0001 |
| 6 | Collagen alpha-6(VI) chain | CO6A6 | COL6A6 | 2.29 | 0.0039 |
| 7 | Tubulin beta-1 chain | TBB1 | TUBB1 | 2.28 | 0.0039 |
| 8 | Collagen alpha-1(III) chain | CO3A1 | COL3A1 | 2.26 | 0.0015 |
| 9 | Decorin | DCN | PGS2 | 2.20 | < 0.0001 |
| 10 | Rho-related GTP-binding protein RhoA | RHOA | RHOA | 2.20 | < 0.0001 |
| 11 | Vimentin | VIM | VIME | 2.16 | < 0.0001 |
| 12 | Annexin A2 | ANXA2 | ANXA2 | 2.16 | < 0.0001 |
| 13 | Cellular retinoic acid-binding protein 1 | CRABP1 | RABP1 | 2.16 | 0.00014 |
| 14 | Protein S100-A9 | S100A9 | S10A9 | 2.14 | 0.0011 |
| 15 | Bisphosphoglycerate mutase | BPGM | PMGE | 2.14 | 0.0065 |
| 16 | Fibrillin-1 | FBN1 | FBN1 | 2.13 | < 0.0001 |
| 17 | Carbonic anhydrase 3 | CA3 | CAH3 | 2.13 | < 0.0001 |
| 18 | Cathepsin G | CTSG | CATG | 2.11 | 0.00084 |
| 19 | Flavin reductase (NADPH) | BLVRB | BLVRB | 2.11 | < 0.0001 |
| 20 | Alpha-1-antitrypsin | SERPINA1 | A1AT | 2.09 | < 0.0001 |
| 21 | IgG receptor FcRn large subunit p51 | FCGRT | FCGRN | 2.08 | 0.0039 |
| 22 | Catalase | CAT | CATA | 2.07 | < 0.0001 |
| 23 | Transmembrane glycoprotein NMB | GPNMB | GPNMB | 2.07 | 0.0065 |
| 24 | Peptidyl-prolyl cis-trans isomerase FKBP10 | FKBP10 | FKB10 | 2.07 | 0.0039 |
| 25 | Band 3 anion transport protein | SLC4A1 | B3AT | 2.02 | < 0.0001 |
| 26 | Thy-1 membrane glycoprotein (Fragment) | THY1 | E9PIM6 | 0.49 | < 0.0001 |
| 27 | Synapsin-2 | SYN2 | A0A087WW96 | 0.49 | < 0.0001 |
| 28 | 2-oxoglutarate dehydrogenase-like, mitochondrial | OGDHL | OGDHL | 0.48 | < 0.0001 |
| 29 | Synaptosomal-associated protein 25 | SNAP25 | SNP25 | 0.48 | < 0.0001 |
| 30 | Brevican core protein | BCAN | PGCB | 0.48 | < 0.0001 |
| 31 | Neuronal-specific septin-3 | SEPT3 | SEPT3 | 0.48 | < 0.0001 |
| 32 | Mothers against decapentaplegic homolog 2 | SMAD2 | MADH2 | 0.48 | 0.0039 |
| 33 | Solute carrier family 2, facilitated glucose transporter member 14 | SLC2A14 | GTR14 | 0.47 | 0.00035 |
| 34 | Tubulin alpha-4A chain | TUBA4A | TBA4A | 0.47 | < 0.0001 |
| 35 | Opioid-binding protein/cell adhesion molecule | OPCML | OPCM | 0.47 | < 0.0001 |
| 36 | Solute carrier family 12 member 5 | SLC12A5 | S12A5 | 0.47 | < 0.0001 |
| 37 | Neuronal membrane glycoprotein M6-b | GPM6B | B7Z613 | 0.47 | < 0.0001 |
| 38 | Serine/threonine-protein phosphatase 2B catalytic subunit alpha isoform | PPP3CA | PP2BA | 0.46 | < 0.0001 |
| 39 | Guanine nucleotide-binding protein subunit gamma | GNG2 | G3V2N0 | 0.46 | 0.00035 |
| 40 | Neurofilament light polypeptide | NEFL | NFL | 0.45 | < 0.0001 |
| 41 | Tubulin beta-4A chain | TUBB4A | TBB4A | 0.45 | 0.00035 |
| 42 | Protein kinase C and casein kinase substrate in neurons protein 1 | PACSIN1 | PACN1 | 0.44 | < 0.0001 |
| 43 | Neuromodulin | GAP43 | NEUM | 0.44 | < 0.0001 |
| 44 | Synaptophysin | SYP | SYPH | 0.44 | 0.0054 |
| 45 | Bone morphogenetic protein 4 | BMP4 | BMP2B | 0.44 | < 0.0001 |
| 46 | Synapsin-1 | SYN1 | SYN1 | 0.44 | < 0.0001 |
| 47 | Tubulin polymerization-promoting protein | TPPP | TPPP | 0.44 | < 0.0001 |
| 48 | Synaptogyrin-3 | SYNGR3 | SNG3 | 0.44 | 0.0039 |
| 49 | Visinin-like protein 1 | VSNL1 | VISL1 | 0.43 | < 0.0001 |
| 50 | Excitatory amino acid transporter 2 | SLC1A2 | EAA2 | 0.41 | < 0.0001 |
| 51 | Synaptic vesicle glycoprotein 2A | SV2A | SV2A | 0.41 | < 0.0001 |
| 52 | Guanine nucleotide-binding protein G(o) subunit alpha | GNAO1 | GNAO | 0.41 | < 0.0001 |
| 53 | Tubulin beta-3 chain | TUBB3 | TBB3 | 0.41 | < 0.0001 |
| 54 | Hyaluronan and proteoglycan link protein 2 | HAPLN2 | HPLN2 | 0.39 | < 0.0001 |
| 55 | Neuronal membrane glycoprotein M6-a | GPM6A | GPM6A | 0.39 | < 0.0001 |
| 56 | Cell adhesion molecule 2 | CADM2 | CADM2 | 0.38 | < 0.0001 |
| 57 | Ephrin type-A receptor 4 | EPHA4 | E9PG71 | 0.37 | 0.0039 |
| 58 | Proline-rich transmembrane protein 2 | PRRT2 | PRRT2 | 0.34 | < 0.0001 |

| Supplementary Table 2 | | | |  | |  | |  | |  | |  | |
| --- | --- | --- | --- | --- | --- | --- | --- | --- | --- | --- | --- | --- | --- |
| Clinical information of another six BAVM patients | | | | | |  | |  | |  | |  | |
| Sex | Age (years) | Ruptured/unruptured | Location | | Size (cm) | | Seizure history | | S-M grade | | Diffuse/compact | |  |
| Male | 26 | Ruptured | Temporal lobe | | 5.42 | | No | | III | | Compact | |  |
| Female | 35 | Unruptured | Frontal lobe | | 4.49 | | NO | | II | | Compact | |  |
| Male | 28 | Unruptured | Temporal lobe | | 5.01 | | NO | | III | | Compact | |  |
| Female | 25 | Unruptured | Temporal lobe | | 4.84 | | YES | | III | | Diffuse | |  |
| Female | 19 | Unruptured | Frontal lobe | | 4.71 | | NO | | III | | Diffuse | |  |
| Male | 33 | Unruptured | Occipital lobe | | 5.81 | | NO | | III | | Diffuse | |  |
